# No culture? No problem: Clinical utility and pitfalls of non-culture diagnostics for pneumococcal parapneumonic effusions

**DOI:** 10.3389/fmed.2026.1707777

**Published:** 2026-01-15

**Authors:** Ilias E. Dimeas, Georgios-Andreas A. Kiousis, Cormac McCarthy, Zoe Daniil

**Affiliations:** 1 Department of Respiratory Medicine, Faculty of Medicine, University of Thessaly, Biopolis, Larissa, Greece; 2 School of Medicine, University College Dublin, Dublin, Ireland; 3 Department of Respiratory Medicine, St. Vincent’s University Hospital, Dublin, Ireland

**Keywords:** *Streptococcus pneumoniae*, parapneumonic effusion, empyema, non-culture diagnostics, urinary antigen test, pleural fluid antigen detection, polymerase chain reaction (PCR), next-generation sequencing (NGS)

## Abstract

Community acquired pneumonia is the primary cause of hospital admission and the most common infectious cause of mortality in developed countries, with parapneumonic effusion and empyema representing frequent complications. Because *Streptococcus pneumoniae* is the leading microbial cause, accurate diagnostic methods with high sensitivity and specificity are crucial to guide effective, narrower-spectrum anti-microbial therapy. This narrative review analyzes culture-independent methods for the diagnosis of pneumococcal parapneumonic effusions, including urinary antigen detection, pleural fluid antigen detection, and polymerase chain reaction, comparing their performance and highlighting key advantages and limitations. While cultures of blood, sputum, and pleural fluid remain the diagnostic gold standard, they have low sensitivity, are time-consuming, and are often affected by prior antibiotic exposure. Non-culture methods provide faster and sometimes more sensitive alternatives: urinary and pleural fluid antigen tests are rapid and practical but risk false positives and negatives, whereas polymerase chain reaction-based techniques offer high specificity and serotype identification but remain costly and less widely available. Emerging approaches such as proteomics and next-generation sequencing may expand diagnostic capabilities in the future. Culture-independent methods therefore hold clinical value in culture-negative cases and can complement traditional techniques, but cultures remain essential for confirmation and antibiotic susceptibility testing, underscoring the need for further adult-focused studies.

## 1 Introduction

One of the most common complications of pneumonia is the development of pleural effusion (1). It is estimated that this condition occurs in 40% of community acquired pneumonias treated in a hospital setting and about 2%–3% of all pneumonias (2). Parapneumonic effusion is defined as an exudative pleural effusion that develops in the setting of lung infection (3). In the United States, it is estimated that parapneumonic effusions affect 1 million patients yearly (4). It is distinguished into uncomplicated, in which no microbe is detected during Gram staining microscopy, pleural fluid culture is negative, the type of fluid is polymorphonuclear exudate, fluid glucose is >60 mg/dL and fluid pH exceeds 7.20. In case at least one of the above criteria is not fulfilled, it is defined as a complicated parapneumonic effusion, a condition which requires drainage (5). Also, it is estimated that 5%–10% of hospitalized patients finally develop empyema, a condition which is defined as accumulation of purulent fluid into the pleural cavity (3, 4). Empyema is a serious complication of bacterial pneumonia, which requires surgical intervention in up to 30% of cases, and is fatal in 15% of cases (6). Common surgical interventions include decortication, thoracentesis and chest tube drainage. According to the American Thoracic Society (ATS) and the Infectious Diseases Society of America (IDSA) (1), it is strongly recommended for any pleural effusion found in patients with pneumonia or unexplained sepsis with inadequate response to appropriate antimicrobial therapy, that fluid be collected and sent for analysis. Pleural fluid cultures, although still considered as a diagnostic reference method, fail to reveal the pathogen in over 60% of cases (6). Thus, the investigation of empyemas and parapneumonic effusions with other culture-independent methods is of essential clinical importance, as it may enhance antibiotic stewardship and enable physicians to select more efficient therapeutic plans.

*Streptococcus pneumoniae* is the leading microbial cause of parapneumonic effusions and empyema (7) and a major cause of community acquired pneumonia (2). Despite advances in antimicrobial therapy, diagnostics, and vaccination strategies, community-acquired pneumonia remains a major cause of morbidity and mortality in adults worldwide, with *S. pneumoniae* continuing to play a dominant etiological role across regions (8). Timely and precise identification of *S. pneumoniae* is clinically important because it allows for targeted use of narrow-spectrum antibiotics, potentially reducing costs, adverse effects, and the development of resistance. *Streptococcus pneumoniae* has already developed resistance to various antibiotic categories, including macrolides, penicillin, fluoroquinolones and sulfamethoxazole-trimethoprim (9). Therefore, the choice of more targeted therapy over empirical therapy is considered necessary, so that we still have effective therapeutic options at our disposal (10). As a result, in recent years, there has been emerging scientific interest in culture-independent diagnostic methods for parapneumonic effusions and empyema, as cultures often fail to identify the causative microorganism. Beyond parapneumonic effusions, pneumococcus also causes meningitis, sepsis, and upper respiratory infections such as sinusitis and otitis media (11), but the present review focuses specifically on pleural involvement. This article is a narrative review, which aims to present and compare culture-independent methods used for the diagnosis of pneumococcal parapneumonic effusions, as well as to provide a critical analysis of their drawbacks and pitfalls, including cost, required time, availability, potential for underdiagnosis or overdiagnosis, and the risk of unnecessary treatment.

## 2 Methods

This review was conducted as a narrative synthesis in accordance with the Scale for the Assessment of Narrative Review Articles (SANRA) guidelines. A non-systematic literature search was performed in PubMed and Web of Science to identify relevant English-language publications from 1976 to 2025. Search terms included combinations of: “*Streptococcus pneumoniae*,” “parapneumonic effusion,” “empyema,” “urinary antigen test,” “pleural fluid antigen detection,” “polymerase chain reaction (PCR),” “next-generation sequencing,” and related diagnostic terms. References were selected based on clinical relevance and contribution to understanding culture-independent diagnostics in parapneumonic effusions. No structured inclusion or exclusion criteria, Preferred Reporting Items for Systematic reviews and Metanalyses (PRISMA) methodology, or risk-of-bias assessment were applied, consistent with the narrative review design. This approach allows a broad overview of available evidence but may be subject to selection bias.

## 3 Culture-negative parapneumonic effusions: why do cultures fail?

The definitive diagnosis of pneumococcal infection in pleural disease is confirmed by the isolation of the pneumococcus in biological fluids, which are sterile under normal conditions (12). Such fluids include blood and pleural fluid. Cultures provide the possibility of testing the sensitivity of the microbe to several antibiotics. However, the isolation of the microbe in cultures is a time-consuming method (2), which often gives false-negative results. These can be attributed to the small sample size, the conditions of transport and preservation of the samples, the previous administration of empirical antibiotic regimens, a fairly common practice in clinical settings, and the production of autolysin, an enzyme produced by the microbe itself, which induces its lysis (13). Blood cultures have a particularly low rate of positivity (<10%) (1, 14), but pleural fluid cultures also have low sensitivity, estimated to be positive in 30% of cases (1, 15). In pediatric studies, isolation rates are even lower, ranging between 18% and 33% (11). Sputum cultures may sometimes identify pneumococcus, but their diagnostic value is limited by poor sample quality and low specificity due to frequent nasopharyngeal carriage (14). In pleural fluid specifically, false negatives are further explained by the typically low bacterial burden, prior or ongoing antibiotic exposure at the time of thoracentesis, and the ability of pneumococcus to form biofilms within the pleural space, which hinders growth in culture media (16–18). Consistent with these findings, a large prospective multicenter study of hospitalized adults with community-acquired pneumonia demonstrated that no pathogen was identified in the majority of cases despite extensive use of culture (19). As a result, although cultures are still regarded as the diagnostic gold standard, their clinical utility is limited by low sensitivity and delayed results, often necessitating empirical broad-spectrum antibiotic use. This may increase costs, side effects, resistance development, and in inadequately treated cases, progression to complications such as empyema or septicemia. Since molecular diagnostic approaches do not depend on bacterial viability, they are less affected by prior antibiotic exposure and may therefore offer advantages over culture for both clinical diagnosis and epidemiological surveillance (20).

## 4 Non-culture diagnostic techniques: strengths, limitations, and clinical realities

### 4.1 Urinary antigen test (UAT)

The first culture-independent method considered is the rapid detection of pneumococcal antigen in urine. Urinary antigen testing is non-invasive and can be rapidly performed at the bedside, making it useful for initial screening. The most widely used assay is the BinaxNOW immunochromatographic test, which detects the soluble pneumococcal polysaccharide C antigen using specific antibodies. According to the manufacturer, the test has a sensitivity of 86% and a specificity of 94%, based on retrospective data (21). However, reported sensitivity in the literature ranges from 60% (22, 23) to 94% (24), while specificity remains consistently high (90%–100%) (25, 26). A 2013 meta-analysis of 27 studies found a pooled sensitivity of 74% (95% CI: 66.6%–82.3%) and specificity of 97% (95% CI: 92.5%–99.8%) (27), while a second meta-analysis of 10 studies reported sensitivity and specificity of 75 and 95%, respectively (28). Because polysaccharide C is a cell wall component of all pneumococcal strains, the test detects pneumococcal infection but does not provide serotype information (22, 29). Although experimental serotype-specific assays have been described (30), these remain research tools without clinical application.

The probability of a positive UAT result also depends on clinical and laboratory factors such as female sex, tachycardia ≥125 bpm, hypotension, hypoxemia, pleuritic chest pain, the presence of pleural effusion, and elevated blood urea nitrogen ≥30 mg/dL (22). Advantages of the UAT include rapid turnaround (<15 min), non-invasive sampling, ease of performance, and independence from prior antibiotic exposure. Its estimated direct cost is $17 per test, although the effective cost per positive case has been calculated at ~$425 due to the relatively low positivity rate (23, 29, 31–33). Limitations include false positives in patients recently infected, vaccinated with polysaccharide vaccine within the previous 48 h (29, 31), or up to 5 days prior (34). Cross-reactions with other streptococci such as *Streptococcus mitis* may also occur (6, 35, 36). In children, frequent nasopharyngeal carriage of pneumococcus results in a high rate of false positives (10, 36). False negatives also occur because of suboptimal sensitivity (29, 37). Although the test has been suggested to facilitate more targeted antimicrobial therapy and stewardship, its real-world impact on reducing costs or length of stay remains uncertain (29). In addition, differences between commercial assays have been described (38).

Overall, the UAT is a rapid and convenient diagnostic tool, particularly in patients with severe community acquired pneumonia and prior antibiotic exposure, but its limitations in sensitivity, specificity in certain populations, and inability to provide serotype or resistance data mean it should be used only as a complementary test rather than a standalone diagnostic method.

### 4.2 Pleural fluid antigen detection

The second diagnostic method assessed in this review is the detection of pneumococcal antigen in pleural fluid samples. This method exploits the fact that parapneumonic effusion is a common complication of pneumococcal pneumonia and therefore, pleural fluid is an alternative biological sample to urine for the detection of polysaccharide C. Pleural fluid sampling is inherently more invasive than urine collection and is performed only when clinically indicated, such as during diagnostic or therapeutic thoracentesis, or chest tube insertion for parapneumonic effusion or empyema. In this context, antigen testing of pleural fluid is not an additional procedure, but an adjunct analysis of material already obtained as part of standard care. The greater invasiveness of pleural sampling restricts its use to selected patients but enhances diagnostic specificity by directly assessing the infected compartment. It may be particularly useful when urine collection is impractical or in patients already on antibiotics. The main methods for its detection are immunochromatography (ICT) and counter-immunoelectrophoresis (CIE).

In adult patients with pleural effusions (14), immunochromatographic detection of pneumococcal C antigen with the BinaxNOW test showed a sensitivity of 70.6% and a specificity of 93.3%. Andreo et al. (15), in a study of 91 pleural fluid samples from adults, reported ICT sensitivity of 79% and specificity of 93.6% and highlighted situations where urine sampling is difficult (e.g., oliguria, renal disease, reduced conscious-ness, psychiatric illness). In such cases, pleural fluid testing may be advantageous. Herrero et al. (2), in a study of 77 patients (40.3% children), reported a sensitivity of 92.3% but lower specificity (80%), attributed to cross-reactions with Parvimonas spp. In pediatric studies, Flores et al. (13) reported pleural ICT sensitivity and specificity of 88 and 71%, respectively, while Torres et al. (39) found very high values using latex antigen detection (96% sensitivity, 100% specificity). For CIE, older data such as Lampe et al. (40) showed favorable results compared with conventional methods.

Pleural fluid antigen detection has advantages: it enables diagnosis when urine cannot be obtained, can support assessment of whether a pleural effusion is parapneumonic in origin, remains reliable despite prior antibiotics, is compatible with automated analyzers for objectivity; such as quantitative immunoassay platforms used in clinical microbiology laboratories, provides results within <20 min, and in some series, shows higher sensitivity than urine ICT (2, 13). However, false positives occur due to cross-reactions with non-pneumococcal streptococci (e.g., viridans, anaerobes) (6), while false negatives may result from low bacterial load, limited serotype coverage, and modest sensitivity in some series (41, 42). Overall, current evidence supports pleural antigen testing as a useful adjunct to culture, but adult data is sparse, and further studies are needed to establish its role in routine practice.

### 4.3 PCR-based techniques

The third culture-independent method assessed in this review is the PCR, which detects the genetic material of the microorganism. In general, it offers accurate and relatively rapid diagnosis in patients previously exposed to antimicrobials and in culture-negative disease and is used mainly for detecting viruses and atypical microbes in biological samples (43). Various *S. pneumoniae* genes have been targeted, including lytA (autolysin, highly specific for pneumococcus), piaB (ABC transporter permease, linked to iron uptake and virulence) (44), ply (pneumolysin) and psaA (adhesin A), with newer targets such as Spn9802, Xisco, and SP2020 (45).

Reported diagnostic accuracy varies widely across studies (44–49), reflecting heterogeneity in assay targets, specimen types, and reference standards. In adults with community-acquired pneumonia, Bjarnason et al. (43) reported oropharyngeal lytA quantitative real-time PCR sensitivity of 87% (rising to 92% after excluding prior-antibiotic cases) and specificity of 79% against a composite reference (culture ± urinary antigen). Van Schaik et al. (49) evaluated quantitative lytA PCR on oropharyngeal swabs, reporting an area under the curve (AUC) of 0.714 with sensitivity 57.1% and specificity 85.7% at a cutoff of 6 × 10^3^ copies/millilitre, which improved to 72.7 and 84.6% (AUC 0.787) in the subgroup with a complete diagnostic work-up. Gillis et al. (50) compared nasopharyngeal lytA plus cpsA against a composite standard (urine antigen and/or sputum or blood culture), yielding sensitivities around 35% and specificities 98–99%, emphasizing that this was not a direct nasopharyngeal culture comparison. Domínguez et al. (51) conducted PCR on serum samples and detected pneumococcal deoxyribonucleic acid (DNA) in 26.6% overall, corresponding to 34.8% sensitivity among bacteraemic cases only, while non-bacteraemic pneumococcal pneumonia remained uniformly negative, underscoring the sample-type limitation of serum PCR. High performance has been reported in pleural fluid: Falguera et al. (52) using nested ply PCR and Sanz et al. (46) with multiplex lytA, plyA, and psaA assays both achieved high sensitivity and specificity, while Pizzutti et al. (53) demonstrated the feasibility of lytA + penicillin-binding protein 2B gene (pbp2b) quantitative real-time PCR for both detection and *β*-lactam-susceptibility inference in culture-negative paediatric samples. Overall, variability in sensitivity and specificity largely reflects differences in gene targets, cut-offs, specimen type (nasopharyngeal/oropharyngeal swab, serum, or pleural fluid), prior antibiotic exposure, and the composite reference standards used across studies, rather than intrinsic assay unreliability.

Lower specificity is mainly due to cross-reactions with related streptococci such as *S. mitis* and *S. pseudopneumoniae* (43, 46). Ahlers et al. (45) recommend SP2020 and Xisco targets to improve specificity, particularly in colonization-prone upper-airway samples. Sensitivity is also influenced by disease severity, timing of sample collection, comorbidities, immunosuppression, and prior antibiotics (43, 50, 51). Additional limitations include high cost, storage-related variability (e.g., freeze–thaw cycles), and logistic factors influencing turnaround time (51).

Nevertheless, PCR offers the advantage of serotyping, with Gillis et al. (50) reporting serotype identification in 88.2% with lytA and 93.9% with cpsA, thereby supporting epidemiology and vaccine development (46, 54). Such serotype-level data are particularly important in the post-vaccination era, where serotype replacement may substantially influence disease burden and vaccine policy (55). In clinical practice, PCR should be viewed as complementary to cultures: it can identify pneumococcus when cultures fail, but cultures remain necessary for confirming diagnosis and providing antibiotic susceptibility data.

## 5 Direct diagnostic comparisons: non-culture methods versus traditional culture

### 5.1 ICT vs. cultures

As mentioned above, ICT test for pneumococcal detection is a rapid test that provides results in ~15 min (41). It can be applied to both urine and pleural fluid samples, remaining useful even in patients already on antibiotics (23, 29, 31–33). In adults, reported sensitivity ranges from 60% to 94%, and specificity is very high (90%–100%). However, specificity is lower in children due to frequent colonization, positive results may persist weeks after infection, and cross-reactions with other streptococci are well described (6, 35, 36). The test also provides no information on antimicrobial susceptibility. By contrast, cultures from blood or pleural fluid remain the reference method, as they both confirm diagnosis and provide antibiogram data to guide targeted treatment. Their limitations are low sensitivity (10%–30% in blood, <30% in pleural fluid) (14–18), reduced yield after prior antibiotics (29), and slower turnaround (>24–48 h) (2). In summary, ICT provides rapid presumptive diagnosis but lacks resistance data, while cultures remain slower but definitive. The aforementioned could be summarized in Table 1.

**Table 1:** ICT versus culture for pneumococcal diagnosis.

Diagnostic method	ICT	Cultures
Sensitivity	Moderate (60%–94%)	Low (blood: 10%–30% pleural fluid: <30%)
Specificity	High in adults (90%–100%), lower in children due to carriage	Very high (almost 100%)
Duration	15 min	>24–48 h
Prior antibiotic administration	No effect (detects antigen regardless of the presence of dead bacteria)	Affected (require live bacteria)
Antibiotic resistance data	No information	Provide complete phenotypic susceptibility results
Utility	Rapid diagnostic tool, especially in adults with community acquired pneumonia/parapneumonic effusion	Provides definite diagnosis and antibiotic resistance data

Sensitivity and specificity reflect adult data unless stated; culture sensitivity varies by specimen (blood versus pleural fluid).

### 5.2 PCR vs. cultures

PCR generally shows high specificity (79%–100%) (43–45, 47–51), but reported sensitivity varies widely; from as low as 34% (50, 51) to as high as 100% (44, 48). These differences reflect target gene selection and study design: *in vitro* studies report higher values, while *in vivo* sensitivity and specificity are lower (49). PCR is advantageous when organisms are difficult to culture, including *S. pneumoniae* (47). It detects microbial DNA even when bacteria are non-viable, enabling diagnosis after antibiotics and supporting epidemiological studies (56, 57). It also allows serotype distinction (57). Limitations include sensitivity reductions with prior antibiotics (43, 50, 51), higher costs, and variable turnaround times, typically between 4 and 24 h depending on laboratory logistics and the use of automated extraction and amplification systems, as well as cross-reactions with related streptococci (e.g., *S. mitis*, *S. pseudopneumoniae*) (43, 46). Cultures remain the only clinically validated and guideline-endorsed method for comprehensive antimicrobial susceptibility testing and minimum inhibitory concentration (MIC) determination (58, 59). PCR can detect selected resistance genes, such as pbp2b (conferring *β*-lactam susceptibility) and ermB/mef, tetM, cat (genes mediating macrolide, tetracycline, and chloramphenicol resistance), but these molecular results lack broad clinical validation for treatment decisions and currently serve mainly research or surveillance purposes (60). Several Center-for-Disease-Control-and-Prevention (CDC)-standardized multiplex and quadriplex real-time PCR assays include resistance targets such as pbp1a, pbp2b, and pbp2x for *β*-lactams and ermB/mef, tetM, cat for macrolide, tetracycline, and chloramphenicol resistance (3). In pleural fluid, combined lytA + pbp2b quantitative real-time PCR has been explored as an indicator of β-lactam susceptibility (53, 60), but these results cannot be phenotypically confirmed in culture-negative samples. Because β-lactam resistance in *S. pneumoniae* arises from mosaic alterations across multiple penicillin-binding proteins (pbp1a, pbp2b, pbp2x), sequence-based models predicting MICs from isolates remain investigational and are not yet suitable for direct clinical use in pleural-fluid diagnostics (58, 61). Therefore, PCR is best used to complement cultures: it detects pneumococcus when cultures fail, while cultures confirm the diagnosis and provide clinically validated phenotypic susceptibility results that remain essential for guiding therapy. The aforementioned could be summarized in Table 2.

**Table 2:** PCR versus culture for pneumococcal diagnosis.

Diagnostic method	PCR	Cultures
Sensitivity	34%–100% (depends on study)	Low (blood: 10–30% pleural fluid: <30%)
Specificity	High (79%–100%)	Very high (almost 100%)
Duration	4–24 h, depending on platform and workflow	>24–48 h
Prior antibiotic administration	No effect (detects specific genes regardless of the presence of dead bacteria)	Affected (require live bacteria)
Antibiotic resistance data	Detects selected genes; not clinically validated for full susceptibility	Provide complete phenotypic susceptibility results
Utility	Can distinguish different serotypes, not affected by antibiotic administration	Provides definite diagnosis and antibiotic resistance data

Reported PCR sensitivity varies by target gene, specimen type, and study design (*in vitro* vs. *in vivo*). Cultures remain the only source of antimicrobial susceptibility data.

### 5.3 ICT vs. PCR

Based on the above data, it is useful to provide a brief comparison between ICT and PCR. Generally, the first method presents significant advantages in terms of its speed (~15 min), straightforward application in clinical practice, wide availability, and low cost (23, 29, 31–33, 41). However, it has been linked to false-positive results in children due to frequent nasopharyngeal colonization (10, 36). PCR, on the other hand, although it provides accurate diagnosis with potential for serotype identification (50, 57), presents several drawbacks. It is not universally available, is more expensive, requires specialized personnel and equipment, and typically yields results within about 1 day (51). Although this is faster than cultures, it remains significantly longer than ICT. In conclusion, both methods play complementary roles alongside cultures in diagnosing pneumococcal disease. ICT is particularly useful for rapid bedside diagnosis and in resource-limited settings (31), whereas PCR is more valuable in research and epidemiological studies due to its serotyping capacity, which may contribute to vaccine development (50, 54). The aforementioned could be summarized in Τable 3.

**Table 3:** PCR versus ICT: practical comparison in suspected pneumococcal disease.

Diagnostic method	PCR	ICT
Sensitivity	34%–100% (depends on study)	Moderate (60%–94%)
Specificity	High (79%–100%)	High in adults (90%–100%), lower in children due to carriage
Duration	4–24 h, depending on platform and workflow	15 min
Prior antibiotic administration	No effect (detects specific genes regardless of the presence of dead bacteria)	No effect (detects antigen regardless of the presence of dead bacteria)
Antibiotic resistance data	Detects selected genes; not clinically validated for full susceptibility	No information
Utility	Can distinguish different serotypes, not affected by antibiotic administration	Rapid diagnostic tool, especially in adults with CAP/parapneumonic effusion
Cost	High (requires equipment, specialized personnel, limited availability)	Low (highly available)

Values summarize ranges from heterogeneous studies; local performance depends on assay, specimen, and pre-test probability.

## 6 Pediatric experience vs. adult clinical reality: a critical appraisal

At this point, it is necessary to highlight the fact that while a sufficient number of notable studies have been conducted in the pediatric population regarding the aforementioned diagnostic methods (2, 10, 11, 13, 36, 39–41, 48, 53, 56, 57, 62), studies are limited in availability regarding the adult population. For instance, we cite the study by Andreo et al. (15), which is the only one that highlighted the usefulness of pleural fluid ICT in adults. This difference in literature can be attributed to several reasons. First, children, especially those aged <5 years, present more often invasive infections from *S. pneumoniae*, such as pneumonia and meningitis, which also carry a higher clinical significance, as they have been associated with more severe complications that are life-threatening, such as empyema and sepsis (63). This implies a higher number of cases available for research in children than in adults. Furthermore, pneumococcus is a microbe that colonizes the nasopharynx in a >40%–60% of children (64), which makes the pediatric population suitable for conducting studies of transmission and surveillance of serotypes. Another reason that we could attribute this difference in the literature is that children are a population group that is subject to vaccination with conjugate vaccines, therefore they present notable research interest regarding clinical trials of new vaccines (65), the epidemiology of the disease (e.g., selection of serotypes involved in vaccines) and consequently, the application of diagnostic methods that will provide reliable diagnoses. Furthermore, studies of adults tend to focus on cases of high clinical severity and with various comorbidities, such as immunosuppression, chronic lung diseases, renal diseases and hematological malignancies (66). Generally, in adults with CAP, it is recommended to initiate antimicrobial treatment empirically, based on guidelines from recognized organizations (1, 67), without any recommendation for further diagnostic investigation. Thus, only a few adults are diagnosed with confirmed pneumococcal infection and the selection of representative samples for studies is more laborious than in pediatric population, which tends to be investigated more thoroughly, due to the high research interest mentioned above.

This gap highlights that pediatric evidence cannot be directly extrapolated to adults, underscoring the need for broader studies in the latter. A large percentage of children, especially those aged <6 years, are carriers of *S. pneumoniae* in the nasopharynx. Reported colonization rates range between 34% and 53% (64, 68), while in adults the corresponding rate is approximately 4%, although detailed data remain scarce. Colonization is influenced by several factors, including young age (with up to 65% carriage in 2–3-year-olds), contact with siblings, daycare attendance, recent antibiotic use, and preceding respiratory infections (68). Colonization predisposes to pneumococcal infection but is also a major source of false-positive results in the urinary antigen test in children. While in adults this test is highly specific (>95%) (29, 51), specificity in children falls below 70% (10, 29), with one study reporting a false-positive rate of 15% (62). Sputum culture is also difficult for children, limiting its role largely to adult populations. Beyond these diagnostic issues, bibliographic data is more abundant in pediatrics than adults (69, 70). This reflects both higher colonization rates in children and the development of conjugate vaccines primarily for pediatric use, as *S. pneumoniae* causes severe infections in this group (meningitis, pneumonia, otitis media). In adults, the low colonization rate necessitates larger population studies to achieve statistical power. Existing studies focus mainly on severe disease (hospitalized pneumonia, sepsis), leaving gaps in epidemiology and colonization data. Given these differences, additional clinical studies in adults are required to draw more reliable conclusions about the sensitivity and specificity of current diagnostic methods in this population.

## 7 Diagnostic stewardship and clinical guidelines: navigating the gray zone

According to the official European Respiratory Society/European Society of Thoracic Surgeons (ERS/ESTS) 2023 guidelines (67), no explicit recommendation exists for the use of culture-independent methods in parapneumonic effusions or empyema due to *S. pneumoniae*. These guidelines emphasize sterile pleural puncture under ultrasound guidance using a small-bore tube, measurement of pH, glucose, and lactate dehydrogenase and sending pleural fluid samples for Gram stain microscopy and culture, along with blood cultures. The RAPID score (71) (Table 4) is highlighted as a prognostic indicator of mortality, incorporating age, blood urea nitrogen, purulence, infection source, and serum albumin.

**Table 4:** The RAPID score as a prognostic indicator for mortality in patients with pleural infection.

Parameter	0 points	1 point	2 points
Renal (blood urea nitrogen)	<30 mg/dL (<5 mmol/L)	30–48 mg/dL (5–8 mmol/L)	>48 mg/dL (>8 mmol/L)
Age	<50 years	50–70 years	>70 years
Purulence of pleural fluid	Non-purulent	Purulent (pus present)	—
Infection source	Community acquired	Hospital-acquired	—
Dietary (serum albumin)	≥27 g/L	<27 g/L	—
Scoring and prognostic groups	Low risk: 0–2 points	Medium risk: 3–4 points	High risk: 5–7 points

Adapted from Rahman et al. (71).

In cases of complicated effusion or inadequate drainage, combined intrapleural recombinant tissue plasminogen activator and deoxyribonuclease therapy is recommended, with surgical evaluation by video-assisted thoracoscopic surgery if this approach fails. Empirical antibiotics should be initiated to cover common community-acquired-pneumonia pathogens such as third-generation cephalosporin or amoxicillin/clavulanate and then adjusted according to cultures and antibiogram, for a total of 2–4 weeks. For pneumococcal effusions specifically, the principle remains the same, with the choice of narrower-spectrum agents once susceptibility is known.

The ATS/IDSA 2019 guidelines (1) likewise do not provide specific recommendations for culture-independent diagnostics. They stress the importance of effusion drainage, fluid analysis, and empirical antibiotic initiation, with escalation to fibrinolysis or surgery if resolution does not occur. Their risk stratification differs, using four levels based on pH, effusion size, microbial detection, and the presence of pus.

Although neither guideline includes culture-independent methods in their algorithms, several studies cited in this review show that ICT (urine, pleural fluid) and PCR may support targeted therapy. Still, high cost, limited availability, and false-positive/negative results restrict their role. In summary, official guidelines remain conservative and do not endorse culture-independent diagnostics for daily clinical practice.

## 8 Limitations and real-world considerations: selection Bias and generalizability

This review and the studies included are subject to several limitations. Since the COVID-19 pandemic, the conduct of new studies on *S. pneumoniae* diagnostics has been limited, so most available data derives from older cohorts. Many were retrospective (2, 14), which introduces information and selection bias, reduced control of confounders (e.g., prior antibiotic intake, timing of sample collection), and variability in how data were recorded. Furthermore, several studies had small sample sizes (26, 33, 39, 46, 49) and restricted geographic coverage (23, 30, 33). Methodological weaknesses commonly reported included selection and publication bias, comparison with low-sensitivity reference methods, and confounding due to storage conditions, previous antibiotic use, or exclusion of patients with comorbidities/immunosuppression (24, 27, 28, 33, 38, 42, 50, 51). The study setting also matters: *in vitro* and in silico studies (45, 47, 49, 51) often overestimate sensitivity/specificity under controlled conditions, while human clinical studies provide more realistic estimates but are vulnerable to uncontrolled variables, subjective interpretation, and loss to follow-up. Finally, much of the evidence comes from pediatric cohorts (2, 13, 39), limiting extrapolation to adults, as previously discussed. Another limitation is the absence of clear diagnostic algorithms from ERS and ATS, which currently do not recommend routine culture-independent testing.

## 9 Future directions: emerging technologies and diagnostic horizons

Several emerging technologies show promise but remain largely confined to research settings. Next-generation sequencing (NGS) can be performed as metagenomic (mNGS), which analyzes all microbial genetic material in a sample, or as targeted NGS (tNGS), which amplifies specific primers. Studies report high diagnostic yield in severe pneumonia, particularly when empirical therapy fails or in mixed infections (72–74). NGS can identify resistance and virulence genes and detect pathogens that are difficult or slow to culture (72). Results can be obtained in 9–48 h, but the method remains costly, technically complex, and, especially with mNGS, limited by inability to clearly separate pathogens from colonizers or contaminants (73, 75). Proteomics represents another novel approach, analyzing pneumococcal proteins (secreted, surface, extracellular) with techniques such as mass spectrometry. It may identify diagnostic biomarkers, clarify virulence factors, and support vaccine development (76–78). Samples may include blood, sputum, tracheal aspirates, or swabs (79). Proteomic studies have also shown potential in detecting host-response proteins, such as markers of oxidative stress and complement activation (80). However, the heterogeneity of *S. pneumoniae* strains, each expressing distinct proteins, complicates clinical translation (79). Parallel advances in automation and workflow integration are also improving the practicality of molecular methods such as PCR. Reducing turnaround times from the current 4–24 h toward near-real-time processing could bridge conventional PCR with NGS-based and proteomic workflows, creating hybrid diagnostic pipelines that combine speed with genomic and proteomic depth. Beyond nucleic acid amplification techniques, the detection of cell-free DNA in pleural fluid has demonstrated diagnostic potential in parapneumonic effusions, particularly in culture-negative cases as another alternative culture-independent approach (81). In summary, both NGS and proteomics hold significant research interest and may shape future diagnostics, but further validation, cost-effectiveness studies, and methodological simplification are required before their incorporation into routine clinical practice.

## 10 Conclusions: balancing diagnostic accuracy, clinical utility, and practical realities

In conclusion, the detection of *S. pneumoniae* antigen in urine or pleural fluid samples by immunochromatographic test and PCR represents a useful adjunct in the diagnosis of pneumococcal parapneumonic effusions, as it may support the selection of targeted and narrow-spectrum antimicrobial treatment and help limit the development of antibiotic-resistant strains. ICT is characterized by rapid turnaround, ease of use, and relatively low cost; when performed on pleural fluid it may be particularly helpful if urine collection is not feasible. PCR, in contrast, while offering high sensitivity and specificity with the additional advantage of serotype identification, remains financially demanding, requires specialized resources, and is more time consuming than ICT. Nevertheless, blood and pleural fluid cultures remain indispensable, as they confirm the diagnosis and provide antimicrobial susceptibility data. Because official guidelines do not currently recommend culture-independent methods for routine practice, further large-scale clinical studies, particularly in adults, are required to clarify their role. Evaluating the cost–benefit balance of such methods is essential to avoid unnecessary testing and health system burden, while ensuring that targeted therapy is available to prevent resistance. Emerging technologies such as NGS and proteomics, while not yet ready for routine use, warrant further investigation in research settings to define their future role in diagnosis and management of parapneumonic effusions.
